# Morbidity and Mortality following Surgery for Hip Fractures in Elderly Patients

**DOI:** 10.1155/2019/7084657

**Published:** 2019-02-05

**Authors:** Hebattu-allah E. Zaki, Shereen M. Mousa, Salma M. S. El Said, Ahmed K. Mortagy

**Affiliations:** Department of Geriatrics and Gerontology, Faculty of Medicine, Ain Shams University, Abbassia, Cairo, Egypt

## Abstract

**Aim:**

To determine morbidity and mortality in elderly patients following hip fracture surgery in Egypt and its correlates and to determine the utility of the POSSUM scale to predict morbidity and mortality among our population.

**Methodology:**

We assessed postoperative morbidity and mortality following hip fracture surgery in a 6-month prospective observational study of 100 elderly patients who were undergoing surgical repair at the beginning of the study. The exclusion criteria included surgically unfit patients and patients refusing to participate in the study. The study was conducted in Ain Shams University Hospital, Ain Shams Specialized Hospital, and El-helal Hospital.

**Results:**

The subjects were categorized as survivors and nonsurvivors according to the 6-month mortality, and the groups were compared statistically according to this classification. The observed 6-month mortality was 19.56%. POSSUM had high specificity for predicting 6-month survival (97.3%). A multivariate regression analysis revealed that postoperative admission to the intensive care unit and lack of ambulation were major risk factors associated with the 6-month mortality.

**Conclusions:**

The POSSUM system had high specificity for predicting survivors (97.3%) but failed to predict mortality (sensitivity = 5.6%). The major risks for 6-month mortality are intensive care unit admission and lack of ambulation.

## 1. Introduction

Hip fractures in the elderly are a major public health concern and can lead to considerable mortality and frequent disability [1, 2]. These fractures place a high economic burden on patients, families, and the medical system [3]. Although recent reports indicate that the incidence of hip fractures is remaining stable, there are concerns that the incidence of hip fractures will increase worldwide due to an increase in the aging population [3–5].

Surgical morbidity and mortality can be prevented or diminished by implementing meticulous preoperative assessment, optimization of clinical condition, careful anesthetic and surgical management, and appropriate postoperative support. Prediction of postoperative outcome using various risk scores is quite important since the patient's physiological status indicates to some extent his/her ability to endure the insult of surgery and to recover uneventfully. The Physiological and Operative Severity Score for the Enumeration of Mortality and Morbidity (POSSUM) is used to assess and standardize the quality of care. It is based on 12 physiological variables measured before surgery and on six operative and postoperative variables, with each variable being scored by a four-grade exponential scale as 1, 2, 4, and 8. Although the POSSUM surgical scoring system is an evidence-based scoring system, it has been found that it may overpredict mortality by a factor of two in high-risk patients, a factor of six in low-risk patients (those with a death risk ≤10%), and a factor of seven in very low-risk patients (those with a death risk ≤5%) [6–8].

Some studies have suggested that the patient's age, sex, and preexisting comorbidities are possible determinants or predictors of hip fracture outcomes; various factors such as male gender, age greater than 75 years, and impaired cognitive function have been reported to be associated with an increased risk of mortality after hip fracture [7, 9–11]. In addition, a history of osteoporotic fracture is considered to increase the risk of sustaining a subsequent hip fracture [12], and ambulatory function recovery has been reported to be primarily dependent on ambulation status before surgery [13]. Many of the reports issued, however, are less clear about the roles and relative contributions of these variables to the outcome. This may be related to study limitations, such as a retrospective design, patient selection bias, the inability to distinguish baseline comorbidities from in-hospital complications, or suboptimal statistical methods. Hip fractures are common and costly. Therefore, it is important to properly identify independent correlates of outcomes in elderly patients who are undergoing hip fracture surgery. Thus, the primary aim of this study was to determine morbidity and mortality in elderly patients following hip fracture surgery in Egypt as the available data regarding mortality and its correlates are very scarce. The secondary aim was to determine the utility of the POSSUM scale to predict morbidity and mortality among our population.

## 2. Methodology

### 2.1. Design and Sampling

A 6-month prospective observational study was conducted to assess the postoperative morbidity and mortality in elderly patients following hip fracture surgery in Egypt. The study was conducted in orthopedic inpatient wards at the Ain Shams University Hospital, the Ain Shams Specialized Hospital, and El-helal Hospital in Cairo, Egypt.

We initially recruited 100 male and female patients with hip fractures who were ≥60 years of age and were to undergo surgical repair. Three patients experienced preoperative mortality and were excluded from the study. One patient died from bronchopneumonia that led to respiratory failure, and one patient had a suspected pulmonary embolism. The remaining patient died after the induction of anesthesia.

### 2.2. Study Population

Exclusion criteria included surgically unfit patients and any patient who refused to participate in the study. For patients who met the inclusion criteria, the nursing staff collected the demographic data from medical charts and caregivers, including age, gender, diagnosis, and type of operation.

The following procedures were performed for all the subjects: informed consent was obtained from the patient or the caregiver. Comprehensive geriatric assessment (preoperative and postoperative) was conducted, which included cognitive assessment using the mini-mental status examination (MMSE) [14]. A cutoff point of 24 was used to indicate cognitive impairment [15]. A screening for depression was conducted using the 15-item geriatric depression scale (GDS-15) [16], in which a score ≥5 suggests depression. Assessment of prefracture functional status was done using activities of daily living (ADL) [17] and instrumental activities of daily living (IADL) evaluations [18]. The preoperative pain was assessed by using the numeric rating scale (NRS). The patients were asked to assign their pain a score that ranged from 0 to 10, where 0 indicated no pain and 10 indicated worst pain imaginable [19]. The Physiological and Operative Severity Score for the Enumeration of Mortality and Morbidity (POSSUM) was also used. This scoring system has been validated for the prediction of mortality after hip fracture surgery [20]. The physiological score was assessed preoperatively, and the operative severity score was determined postoperatively [6]. The combined total of the physiological and operative scores was then entered into a logistic regression equation that determined the risk of death [20]. The confusion assessment method (CAM) [21] was used to identify and recognize delirium, and it was assessed postoperatively for determining postoperative delirium. Follow-up by telephone call was conducted at 30 days, 3 months, and 6 months after surgery to record morbidity and mortality.

## 3. Results

To determine the rate of 6-month mortality in elderly Egyptian patients after surgery for hip fracture, we followed up 97 participants recruited from the orthopedic inpatient ward for 6 months. The subjects were categorized as survivors or nonsurvivors according to the 6-month mortality and were compared statistically according to this classification.

### 3.1. Demographic Correlates of 6-Month Mortality after Hip Surgery

Table 1 shows a comparison of patient characteristics for the survivors and nonsurvivors. Mortality was associated with not working (*p*=0.01), using assistive devices (*p*=0.04), and having dementia or visual impairment (*p*=0.006). The inconsistency in the total numbers in Table 1, e.g., the total of 86 participants in the “sex” category, but a total of only 83 in the “Occupation” category and 70 in the “smoking” category, was due to the inability to collect these data and was considered missing data during statistical analysis.

### 3.2. POSSUM Sensitivity and Specificity in Predicting 6-Month Mortality and Morbidity

Follow-up by telephone call was conducted at 30 days, 3 months, and 6 months after surgery to record morbidity and mortality.

Predictions of mortality and morbidity for individual patients were estimated using the following equations, in which *R*1 relates to mortality and *R*2, morbidity:

(1) $$\begin{array}{c} \text{Loge }\frac{R1}{\left(1-R1\right)}=-7.04+\left(0.13\times \text{physiological score}\right)+\left(0.16\times \text{operative severity score}\right),\\ \text{Loge }\frac{R2}{\left(1-R2\right)}=-5.91+\left(0.16\;\times \text{physiological score}\right)+\left(0.19\times \text{operative severity score}\right). \end{array}$$

Predicted mortality was cross-tabulated with observed mortality.

A patient was considered alive if he was found alive at 6 months follow-up.

Table 2 shows the sensitivity and specificity of the POSSUM system in predicting morbidity and mortality. The observed 6-month mortality was 19.56%, and the observed morbidity was 69.56%. POSSUM had high specificity for predicting survivors (97.3%). The system failed to predict mortality (sensitivity = 5.6%) and showed moderate ability to predict morbidity (sensitivity = 59.4%, specificity = 57.1%).

### 3.3. Preoperative and Postoperative Correlates of 6-Month Mortality after Hip Surgery

Table 3 shows that preoperative functional impairment was strongly associated with mortality, specifically for the following functions: bathing (*p*=0.04), continence (*p*=0.03), handling money (*p*=0.003), transportation (*p*=0.001), shopping (*p*=0.003), housekeeping (*p*=0.001), and taking medications (*p*=0.005). The analysis of relevant postoperative complications and management showed that subjects with postoperative delirium, pressure ulcers, and no ambulation experienced statistically significant mortalities (*p*=0.001) (Table 4). Additionally, the data showed that urinary incontinence (*p*=0.03), admission to the intensive care unit (ICU) (*p*=0.02), and lack of rehabilitation (*p*=0.03) were also risk factors. The multivariate regression analysis revealed postoperative ICU admission and lack of ambulation to be the major risk factors associated with 6-month mortality (Table 5).

## 4. Discussion

Hip fractures are one of the most serious problems in elderly, which have not been sufficiently studied in Egypt for the postoperative 6-month mortality rates. The current study highlights the observed 6-month mortality rates and the predicted rates using the POSSUM system, together with the use of comprehensive geriatric assessment to determine risk factors for increasing mortality.

The 6-month mortality rate observed in our cohort was 19.56%. In the literature, mortality rates following hip fractures vary widely. In a study of 192 hip fracture patients with a mean age of 76.9, the six-month mortality rate following surgery was 25%. The deceased patients were significantly older than the patients who survived [22]. In a systematic review of preoperative predictors for mortality following hip fracture surgery that included 75 studies involving 64,316 subjects, the overall 1-month mortality was 13.3% and the 3- to 6-month mortality was 15.8% [23]. Another study by Marta and colleagues reported a mortality rate of 24.1% between the second and sixth months and 29.3% between the sixth and twelfth months following admission for hip surgery [24]. The wide variability between mortality rates including ours may be attributed to the heterogeneity of the studied population, their characteristics, and the postoperative care they had.

The POSSUM system predicted the 1-month morbidity and mortality in hip fracture surgery and showed a high specificity (97.3%) to detect survivors over the 6-month period of follow-up. However, the system failed to detect mortality. The most important risk factor associated with mortality in our participant cohort was functional disability. The increase was evident in the ADL and IADL items and their relation to mortality. The data also showed that functional disability was related to increased mortality and that there was a higher prevalence of mortality among patients using assistive devices (*p*=0.04) and in the unemployed (*p*=0.01). A Canadian population-based study of 1,329 elderly individuals with hip fractures for whom ADL information was available prior to the hip fracture concluded that direct measures of ADL impairment provide additional prognostic information on mortality for older adults with hip fractures [25]. Thus, we strongly recommend preoperative functional assessment to establish the baseline functional level for postoperative rehabilitation outcomes and further studies for its prognostic value.

The most commonly reported morbidities related to 6-month mortality were delirium, pressure ulcers, and delayed ambulance, which are consistent with previous studies. In a study of 603 patients with hip fractures, Kat et al. showed that there was a delirium incidence of 32.2% in patients who died versus 8.8% in those who survived [26]. In a previous study of 269 patients older than 70 years who underwent surgery for proximal femoral fractures, it was found that pressure ulcers developed in 34.2% of patients and that the presence of ulcers in the postoperative period significantly reduced patient survival (*p*=0.037) [27]. Finally, Foss et al. [28] have reported that the cumulative ambulation score is a highly significant predictor of postoperative mortality, suggesting that postoperative ambulation can widely impact mortality. The most important aspect of these morbidities is that they are preventable. Early preventive measures applied through the orthogeriatric team may reduce morbidities and related mortality, yet further studies are needed.

Geriatric syndromes also affect mortality. We found that patients with dementia had significantly higher mortality. In a large meta-analysis that included 1,782 participants, patients with premorbid dementia demonstrated an almost three times greater mortality than patients without dementia [29]. Our multivariate regression analysis of significant risks associated with mortality revealed that ICU admission and lack of rehabilitation were the most influential factors for mortality. Though our study included only a small number of patients and had the limitation of missing data during the follow-up period, it offers a new insight into mortality following 6 months of hip fracture surgery in Egypt. Many risk factors for mortality could be prevented such as postoperative delirium, pressure ulcers, and immobility. We recommend the introduction of the orthogeriatric team for better outcome for our population with hip fracture surgery.

## Figures and Tables

**Table 1:** Clinical and demographic characteristics of survivors and nonsurvivors at 6 months after surgery.

Variable			Survivors		Nonsurvivors		Total		*P* value
			*N*	%	*N*	%	*N*	%	
Sex		Male	33	50.77	12	57.14	45	45	0.62
		Female	32	49.23	9	42.86	41	41	

Occupation		Housewife	23	36.51	10	50.00	33	33	0.001
		Not working	25	39.68	10	50.00	35	35	
		Working	15	23.81	0	0.00	15	15	

Cigarette smoking		Nonsmoker	34	65.38	14	77.78	48	68.57	0.19
		Ex-smoker	6	11.54	3	16.67	9	12.86	
		Current smoker	12	23.08	1	5.56	13	18.57	

Assistive device use		None	48	78.67	10	50.00	58	71.60	0.04
		Cane	12	19.67	8	40.00	20	24.69	
		Others	1	1.64	2	10.00	3	3.70	

Type of fracture		Subcapital	21	35.59	8	40.00	29	36.71	0.56
		Transcervical	1	1.69	1	5.00	2	2.53	
		Basicervical	4	6.78	1	5.00	5	6.33	
		Intertrochanteric	24	40.68	7	35.00	31	39.34	
		Subtrochanteric	9	15.25	2	10.00	11	13.92	
		Periprosthetic	0	0.00	1	5.00	1	1.27	

Type of repair		PFN	5	7.94	2	10.53	7	8.54	0.17
		DHS	18	28.57	4	21.05	22	26.83	
		Bipolar hemiarthroplasty	22	34.92	6	31.58	28	34.15	
		External fixator	0	0.00	2	10.53	2	2.44	
		Thompson hemiarthroplasty	5	7.94	3	15.79	8	9.76	
		ORIF	10	15.87	1	5.26	11	13.41	
		Hemiarthroplasty	1	1.59	1	5.26	2	2.44	
		THR	2	3.17	0	0.00	2	2.44	

Past surgical history		For hip fracture	4	6.15	0	0.00	4	4.65	0.19
		For others	24	36.92	11	52.38	35	40.70	

Medical history		Hypertension	30	46.15	12	57.14	42	48.84	0.38
		DM	27	41.54	12	57.14	39	45.35	0.21
		Visual impairment	21	32.31	14	66.67	35	40.70	0.006
		Osteoarthritis	19	29.23	7	33.33	26	30.23	0.72
		Falls	13	20.00	5	23.81	18	20.93	0.71
		Liver disease	9	13.85	3	14.29	12	13.95	0.96
		Stroke	8	12.31	5	23.81	13	15.12	0.22
		Urinary incontinence	7	10.77	2	9.52	9	10.47	0.87
		Kidney disease	6	9.23	2	9.52	8	9.30	0.97
		Bronchial asthma	5	7.69	0	0.00	5	5.81	0.09
		IHD	3	4.62	1	4.76	4	4.65	0.98
		COPD	3	4.62	0	0.00	3	3.49	0.19
		Osteoporosis	3	4.62	2	9.52	5	5.81	0.43
		Dementia	2	3.08	5	23.81	7	8.14	0.006

PFN = proximal femoral nail; DHS = dynamic hip screw; ORIF = open reduction internal fixation; THR = total hip replacement; DM = diabetes mellitus; IHD = ischemic heart disease; COPD = chronic obstructive pulmonary disease.

**Table 2:** Sensitivity and specificity of POSSUM in predicting 6-month mortality and morbidity.

Mortality			
Predicted	Observed		
	Dead	Alive	Total
Dead	1	2	3
Alive	17	72	89
Total	18	74	92

Morbidity			
Predicted	Observed		
	Complicated	Noncomplicated	Total
Complicated	38	12	50
Noncomplicated	26	16	42
Total	64	28	92

POSSUM did not predict postoperative 6-month mortality, and its sensitivity was 5.6%, yet it has a high specificity for predicting survivors (97.3%). Regarding morbidity, POSSUM proved of a moderate value in predicting morbidity with sensitivity = 59.4% and specificity = 57.1%.

**Table 3:** Relationship between preoperative functional status and 6-month postoperative mortality.

Variable		Survivor		Nonsurvivors		Total		*P*-value
		*N*	%	*N*	%	*N*	%	
Bathing	Independent	54	83.08	11	55.00	65	76.47	0.04
	Assisted	3	4.62	3	15.00	6	7.06	
	Dependent	8	12.31	6	30.00	14	16.47	

Dressing	Independent	55	84.62	13	65.00	68	80.00	0.13
	Assisted	5	7.69	5	25.00	10	11.76	
	Dependent	5	7.69	2	10.00	7	8.24	

Toileting	Independent	61	93.85	15	75.00	76	89.41	0.08
	Assisted	3	4.62	3	15.00	6	7.06	
	Dependent	1	1.54	2	10.00	3	3.53	

Transfer	Independent	62	95.38	16	80.00	78	91.76	0.13
	Assisted	2	3.08	3	15.00	5	5.88	
	Dependent	1	1.54	1	5.00	2	2.35	

Continence	Independent	61	93.85	14	70.00	75	88.24	0.03
	Assisted	2	3.08	4	20.00	6	7.06	
	Dependent	2	3.08	2	10.00	4	4.71	

Eating	Independent	63	96.92	17	85.00	80	94.12	0.18
	Assisted	1	1.54	2	10.00	3	3.53	
	Dependent	1	1.54	1	5.00	2	2.35	

Money handling	Independent	55	84.62	9	45.00	64	75.29	0.003
	Assisted	6	9.23	6	30.00	12	14.12	
	Dependent	4	6.15	5	25.00	9	10.59	

Telephoning	Independent	45	69.23	10	50.00	55	64.71	0.13
	Assisted	13	20.00	4	20.00	17	20.00	
	Dependent	7	10.77	6	30.00	13	15.29	

Transportation	Independent	47	72.31	5	25.00	52	61.18	0.001
	Assisted	7	10.77	6	30.00	13	15.29	
	Dependent	11	16.92	9	45.00	20	23.53	

Shopping	Independent	47	72.31	6	30.00	53	62.35	0.003
	Assisted	6	9.23	5	25.00	11	12.94	
	Dependent	12	18.46	9	45.00	21	24.71	

Preparing meals	Independent	45	69.23	8	40.00	53	62.35	0.07
	Assisted	8	12.31	5	25.00	13	15.29	
	Dependent	12	18.46	7	35.00	19	22.35	

Housekeeping	Independent	45	69.23	5	25.00	50	58.82	0.001
	Assisted	8	12.31	8	40.00	16	18.82	
	Dependent	12	18.46	7	35.00	19	22.35	

Taking medications	Independent	56	86.15	10	50.00	66	77.65	0.005
	Assisted	7	10.77	7	35.00	14	16.47	
	Dependent	2	3.08	3	15.00	5	5.88	

**Table 4:** Postoperative complications and management associated with mortality.

Variable		Survivor		Nonsurvivors		Total		*P* value
		*N*	%	*N*	%	*N*	%	
Delirium	No	56	90.32	8	40.00	64	78.05	0.001
	Yes	6	9.68	12	60.00	18	21.95	

Pressure ulcers	No	48	77.42	3	14.29	51	61.45	0.001
	Yes	14	22.58	18	85.71	32	38.55	

Urinary incontinence	No	35	56.45	6	28.57	41	49.40	0.03
	Yes	27	43.55	15	71.43	42	50.60	

Falls	No	57	91.94	20	95.24	77	92.77	0.60
	Yes	5	8.06	1	4.76	6	7.23	

Postoperative ICU management	No	46	83.64	8	53.33	54	77.14	0.02
	Yes	9	16.36	7	46.67	16	22.86	

Ambulation	No	8	12.31	18	85.71	26	30.23	0.001
	Yes	57	87.69	3	14.29	60	69.77	

Postoperative rehabilitation	No	44	72.13	17	94.44	61	77.22	0.03
	Yes	17	27.87	1	5.56	18	22.78	

ICU = intensive care unit.

**Table 5:** Multivariate regression analysis of different variables significantly related to postoperative 6-month mortality after hip fracture.

	*B*	Standard error	Significance	Odds	95% CI for odds	
					Lower	Upper
Bathing	−0.58	0.67	0.39	0.56	0.150	2.090
Continence	−0.06	1.79	0.97	0.94	0.028	31.640
Transportation	3.85	2.09	0.07	46.98	0.783	2820.39
Shopping	−4.13	2.18	0.06	0.016	0.001	1.14
Housekeeping	−0.88	1.45	0.55	0.42	0.025	7.074
Taking medications	−0.58	1.25	0.64	0.56	0.049	6.39
Money handling	1.74	1.19	0.14	5.79	0.552	58.78
Ambulation	−4.13	1.49	0.006	0.016	0.001	0.29
Postoperative ICU admission	3.32	1.67	0.04	27.68	1.037	7339.29
Complications	−9.31	50.33	0.85	0.001	0.001	6.236
Constant	0.36	0.92	0.69	1.44		

## Data Availability

The data used to support the findings of this study are available from the corresponding author upon request.
